# Supplementary material for: EnzML: multi-label prediction of enzyme classes using InterPro signatures
Source: BMC Bioinformatics. 2012 Apr 25;13:61. doi: 10.1186/1471-2105-13-61 (PMC3483700; doi:10.1186/1471-2105-13-61)
Supplement: Addtional file 5 — The Java code to format the data files, evaluate and predict. The file enzml_java_code.tar.gz contains the Java code used to format database data to ARFF and XML formats, to execute cross and train-test (jackknife) evaluations and to record evaluation results to database. More information is included in the readme.txt file and the Javadoc files. The code can be used with a MySQL database. To use a different database software, other JDBC drivers might be required. [file 1471-2105-13-61-S5.gz › java_code/utils/doc/uk/ac/ed/inf/utils/webutils/XMLUtils.html]

XMLUtils


---


|  |  |  |
| --- | --- | --- |
| **Overview** \| **Package** \| **Class** \| **Use** \| **Tree** \| **Deprecated** \| **Index** \| **Help** | |  |
| **PREV CLASS**   NEXT CLASS | **FRAMES**    **NO FRAMES**     **All Classes** |  |
| SUMMARY: NESTED \| FIELD \| CONSTR \| METHOD | DETAIL: FIELD \| CONSTR \| METHOD |  |


---


## uk.ac.ed.inf.utils.webutils Class XMLUtils

```
java.lang.Object
  uk.ac.ed.inf.utils.webutils.XMLUtils
```

---

``` public class XMLUtils extends java.lang.Object ```

XML utilities. To parse simple XML etc.

**Version:**
:   12 Dec 2007

**Author:**
:   Luna De Ferrari luna.deferrari-at-ed.ac.uk

---

**Constructor Summary**

`XMLUtils()`


| **Method Summary** | |
| --- | --- |
| `static java.lang.String` | `fromDOMtoFile(org.w3c.dom.Document DOMdocument, java.lang.String filePath)`             From JTidy DOM to file |
| `static java.lang.String` | `getXmlComment(java.lang.String text)`             Returns a string formatted as xml comment: |
| `static XmlNode` | `getXmlFromHTML(java.lang.String url, java.lang.String xhtmlFile)` |
| `static XmlNode` | `getXmlFromReader(java.io.BufferedReader reader)`             Get the XML as SimpleElement from a URL reader. |
| `static org.w3c.dom.Document` | `tidyDOM(java.io.InputStream inputStream)`             JTidy is designed to read-in typical-quality (that is to say, bad) HTML and output something cleaner (you have a choice of options), and also provides a DOM interface for traversing HTML documents that can be fed to an XML parser. |

**Methods inherited from class java.lang.Object**

`equals, getClass, hashCode, notify, notifyAll, toString, wait, wait, wait`

**Constructor Detail**

### XMLUtils

```
public XMLUtils()
```


**Method Detail**

### fromDOMtoFile

```
public static java.lang.String fromDOMtoFile(org.w3c.dom.Document DOMdocument,
                                             java.lang.String filePath)
                                      throws java.lang.SecurityException,
                                             java.io.FileNotFoundException
```

:   From JTidy DOM to file

    :   **Parameters:**: `DOMdocument` -: `filePath` - **Returns:**: the file path **Throws:**: `java.lang.SecurityException`: `java.io.FileNotFoundException`

---


### getXmlComment

```
public static java.lang.String getXmlComment(java.lang.String text)
```

:   Returns a string formatted as xml comment:

    ```
     <!--  comment to be inserted -->
    ```

    :   **Parameters:**: `text` - the comment to be inserted **Returns:**: the comment surrounded by appropriate xml-comment markers.

---


### getXmlFromHTML

```
public static XmlNode getXmlFromHTML(java.lang.String url,
                                     java.lang.String xhtmlFile)
                              throws java.io.IOException
```

:   **Throws:**: `java.io.IOException`

---


### getXmlFromReader

```
public static XmlNode getXmlFromReader(java.io.BufferedReader reader)
                                throws java.io.IOException
```

:   Get the XML as SimpleElement from a URL reader. SimpleElement can then be
    used by a SimpleDOMParser.

    :   **Parameters:**: `reader` - **Returns:**: a tree of xml elements **Throws:**: `java.io.IOException`

---


### tidyDOM

```
public static org.w3c.dom.Document tidyDOM(java.io.InputStream inputStream)
                                    throws java.lang.SecurityException,
                                           java.io.FileNotFoundException
```

:   JTidy is designed to read-in typical-quality (that is to say, bad) HTML
    and output something cleaner (you have a choice of options), and also
    provides a DOM interface for traversing HTML documents that can be fed to
    an XML parser.
    http://www.ibm.com/developerworks/xml/library/j-jtp03225.html

    :   **Parameters:**: `inputStream` - **Returns:**: a DOM representation of the HTML document in the input stream **Throws:**: `java.io.FileNotFoundException`: `java.lang.SecurityException`


---


|  |  |  |
| --- | --- | --- |
| **Overview** \| **Package** \| **Class** \| **Use** \| **Tree** \| **Deprecated** \| **Index** \| **Help** | |  |
| **PREV CLASS**   NEXT CLASS | **FRAMES**    **NO FRAMES**     **All Classes** |  |
| SUMMARY: NESTED \| FIELD \| CONSTR \| METHOD | DETAIL: FIELD \| CONSTR \| METHOD |  |


---
